# 

**Published:** 1881-04

**Authors:** 


*Wanted.*—The following numbers of the American Journal of Dental Science. Any one having them or any of them, will confer a great favor on the writer by sending them to

Snowden & Cowman's Dental Depot. Twenty-five cents each will be paid. Vol. V., No. 12. Vol. VI., No. 2. Vol. VI., No. 7. Vol. XI. No. 11.

James B. Hodgkin.

### BIBLIOGRAPHICAL.

### To Readers and Correspondents.

Communications intended for publication, and exchange journals and papers, should be addressed to the EDITOR OF THE AMERICAN JOURNAL OF DENTAL SCIENCE, 259 N. Eutaw St., Baltimore, Md. All letters relating to the business of the journal. or containing cash remittances, to be directed to Messrs. SNOWDEN & COWMAN. Articles for publication should be received by the editor at least three weeks previous to the first of the month on which the number in which it is designed for them to appear, is issued.

### BIBLIOGRAPHICAL.

Elementary Treatise on Practical Chemistry and Qualitative Inorganic Analysis. By Frank Clowes......575
Essentials of Chemistry. Organic and Inorganic. By R. A. Witthaus, A M., M. D......576

### MONTHLY SUMMARY.

Bleaching Gutta Percha......576

☞The American Journal of Dental Science will contain fifty pages of reading matter in each number, making a volume of about *Six Hundred* pages,

EXCLUSIVE OF ADVERTISEMENTS.

THE

### AMERICAN JOURNAL

OF

### DENTAL SCIENCE.

The *Journal* is issued on the first of each month and contains not less than *fifty* pages of reading matter in each number, exclusive of advertisements, making a volume of *six hundred* pages per annum.

In each number will be found Original Communications, Correspondence, Selected Articles, Monthly Summary, Bibliographical Notices and Editorial Matter, and every effort will be exerted to render the *Journal* worthy of the patronage of the Dental profession.

A generous co-operation is respectfully solicited from the members of the profession; and as the *Journal* has now a printing office of its own, which materially lessens the cost of its publication, it will be issued at the reduced subscription price of

**$2.50 Per Annum in Advance.**

Communications are respectfully solicited on all subjects pertaining to the practice of dentistry, as well as reports of cases occurring in dental practice.

### RATES OF ADVERTISING.

| | 1 MO. | 2 MO. | 3 MO. | 6 MO. | 12 MO. |
|---|---|---|---|---|---|
| 1 PAGE. | $15 00 | $22 50 | $30 00 | $50 00 | $100 00 |
| ½ " | 10 00 | 15 00 | 20 00 | 30 00 | 50 00 |
| ¼ " | 7 00 | 11 00 | 15 00 | 20 00 | 30 00 |
| ⅛ " | 5 00 | 8 00 | 11 00 | 15 00 | 20 00 |

All original communications designed for publication, works for review, new instruments for notice, exchanges, &c., should be directed to the editor, 259 N. Eutaw St., Baltimore, Md.

All advertisements and subscriptions should be addressed to

SNOWDEN & COWMAN,

*Publishers of the Am. Journal of Dental Science.*

86 W. FAYETTE ST. BET. CHARLES & LIBERTY STS.,

BALTIMORE, MD.

